# Different Expression Patterns of Genes from the *Exo-Xis* Region of Bacteriophage λ and Shiga Toxin-Converting Bacteriophage Ф24_B_ following Infection or Prophage Induction in *Escherichia coli*

**DOI:** 10.1371/journal.pone.0108233

**Published:** 2014-10-13

**Authors:** Sylwia Bloch, Bożena Nejman-Faleńczyk, Aleksandra Dydecka, Joanna M. Łoś, Agnieszka Felczykowska, Alicja Węgrzyn, Grzegorz Węgrzyn

**Affiliations:** 1 Department of Molecular Biology, University of Gdańsk, Gdańsk, Poland; 2 Department of Microbiology, University of Szczecin, Szczecin, Poland; Institute of Immunology and Experimental Therapy, Polish Academy of Sciences, Poland

## Abstract

Lambdoid bacteriophages serve as useful models in microbiological and molecular studies on basic biological process. Moreover, this family of viruses plays an important role in pathogenesis of enterohemorrhagic *Escherichia coli* (EHEC) strains, as they are carriers of genes coding for Shiga toxins. Efficient expression of these genes requires lambdoid prophage induction and multiplication of the phage genome. Therefore, understanding the mechanisms regulating these processes appears essential for both basic knowledge and potential anti-EHEC applications. The *exo-xis* region, present in genomes of lambdoid bacteriophages, contains highly conserved genes of largely unknown functions. Recent report indicated that the Ea8.5 protein, encoded in this region, contains a newly discovered fused homeodomain/zinc-finger fold, suggesting its plausible regulatory role. Moreover, subsequent studies demonstrated that overexpression of the *exo-xis* region from a multicopy plasmid resulted in impaired lysogenization of *E. coli* and more effective induction of λ and Ф24_B_ prophages. In this report, we demonstrate that after prophage induction, the increase in phage DNA content in the host cells is more efficient in *E. coli* bearing additional copies of the *exo-xis* region, while survival rate of such bacteria is lower, which corroborated previous observations. Importantly, by using quantitative real-time reverse transcription PCR, we have determined patterns of expressions of particular genes from this region. Unexpectedly, in both phages λ and Ф24_B_, these patterns were significantly different not only between conditions of the host cells infection by bacteriophages and prophage induction, but also between induction of prophages with various agents (mitomycin C and hydrogen peroxide). This may shed a new light on our understanding of regulation of lambdoid phage development, depending on the mode of lytic cycle initiation.

## Introduction

Bacteriophage λ, which is a virus infecting *Escherichia coli* cells, has been used as a model in studies in the fields of microbiology and molecular biology for over 6 decades (for a review, see [1]). Shortly after its discovery, it appeared that there are other bacteriophages, which genomes’ organization and developmental pathways are similar to those of λ, therefore, the family of lambdoid bacteriophages has been formally established. One of characteristic features of these viruses is their ability to follow two alternative developmental pathways. The lytic pathway includes phage genome replication and synthesis of phage-encoded regulatory and structural proteins, leading to production and liberation of progeny virions. The lysogenic pathway consists of integration of the phage genome into host chromosome, forming a prophage, and a passive replication of this form of the viral genome together with bacterial DNA (host cells bearing integrated phage genomes are called lysogens). However, the lysogenic stage is not permanent. A developmental switch, which consists of prophage induction, excision of phage DNA from the host chromosome, and initiation of the lytic mode of phage development, can occur under certain conditions causing a DNA damage in the host cell (for reviews, see [2], [3]).

Genomes of some lambdoid bacteriophages, apart from genes characteristic for the whole family, contain also genes (abbreviated *stx*) encoding Shiga toxins. If *E. coli* is lysogenic with such a phage, it may be highly pathogenic to humans. Bacterial strains bearing such prophages are called Shiga toxin-producing *E. coli* (STEC), and this group includes enterohemorrhagic *E. coli* (EHEC) strains that are particularly dangerous pathogens [4]–[6]. Bacteriophages bearing *stx* genes in their genomes are known as Shiga toxin-converting bacteriophages or Stx phages [7]–[9]. The recent outbreak that occurred in Germany in 2011 resulted in over 4,000 symptomatic infections, including over 50 fatal cases. This can be an indication of severity of STEC-mediated infections and significance of medical problems caused by bacteria lysogenic with these phages [10]–[14].

In this light, detailed understanding the mechanisms of regulation of lambdoid phages’ development appears crucial, particularly because Stx prophage induction and effective replication of the phage genome are indispensible for efficient production of Shiga toxins [15]–[18]. This stems from the fact that expression of majority of genes of lambdoid prophages, including *stx* genes in Stx prophages, is strongly inhibited by the phage-encoded cI repressor (despite the fact that this repression may be weaker in at least some Stx phages than in λ) [2], [3], [19]. The prophage induction may be either spontaneous (but it occurs with a low frequency) or caused by factors and agents provoking DNA lesions, thus provoking the bacterial S.O.S. response which indirectly results in cleavage of the cI protein and de-repression of most of phage genes (for reviews, see [2], [3]). In the case of Shiga toxin-converting prophages, among such factor and agents there are UV irradiation, antibiotics interfering with DNA metabolism (like mitomycin C), and hydrogen peroxide (which appears to be the most plausible compound causing induction of Stx prophages) [20], [21]. However, despite determination of molecular principles of cI-mediated regulation of gene expression, we are still far from complete understanding of mechanisms influencing efficiency of lambdoid prophage induction and its further lytic development.

One mystery in lambdoid phage biology is the *b* region in the viral genome. It is dispensable for phage development under standard laboratory conditions (a phenomenon which is unusual among viruses), but contains an evolutionarily conserved fragment, located between *exo* and *xis* genes and transcribed from the *p*_L_ promoter. This fragment is called the *exo-xis* region, and consists of several open reading frames which functions in phage development are largely unknown. Previous studies demonstrated that overexpression of genes from the *exo-xis* region caused impairment lysogenization of *E. coli* by bacteriophage λ [22]. Subsequent report indicated that the presence of multiple copies of these genes on plasmids positively influenced efficiencies of induction of prophages λ and φ24_B_, one of Shiga toxin-converting phages [23]. Interestingly, it was also found that two orthologs of the λ Ea8.5 protein, encoded by a gene located between *exo* and *xis*, contain a fused homeodomain/zinc-finger fold [24]. This strongly suggest a regulatory role of this protein.

**Figure 1:**
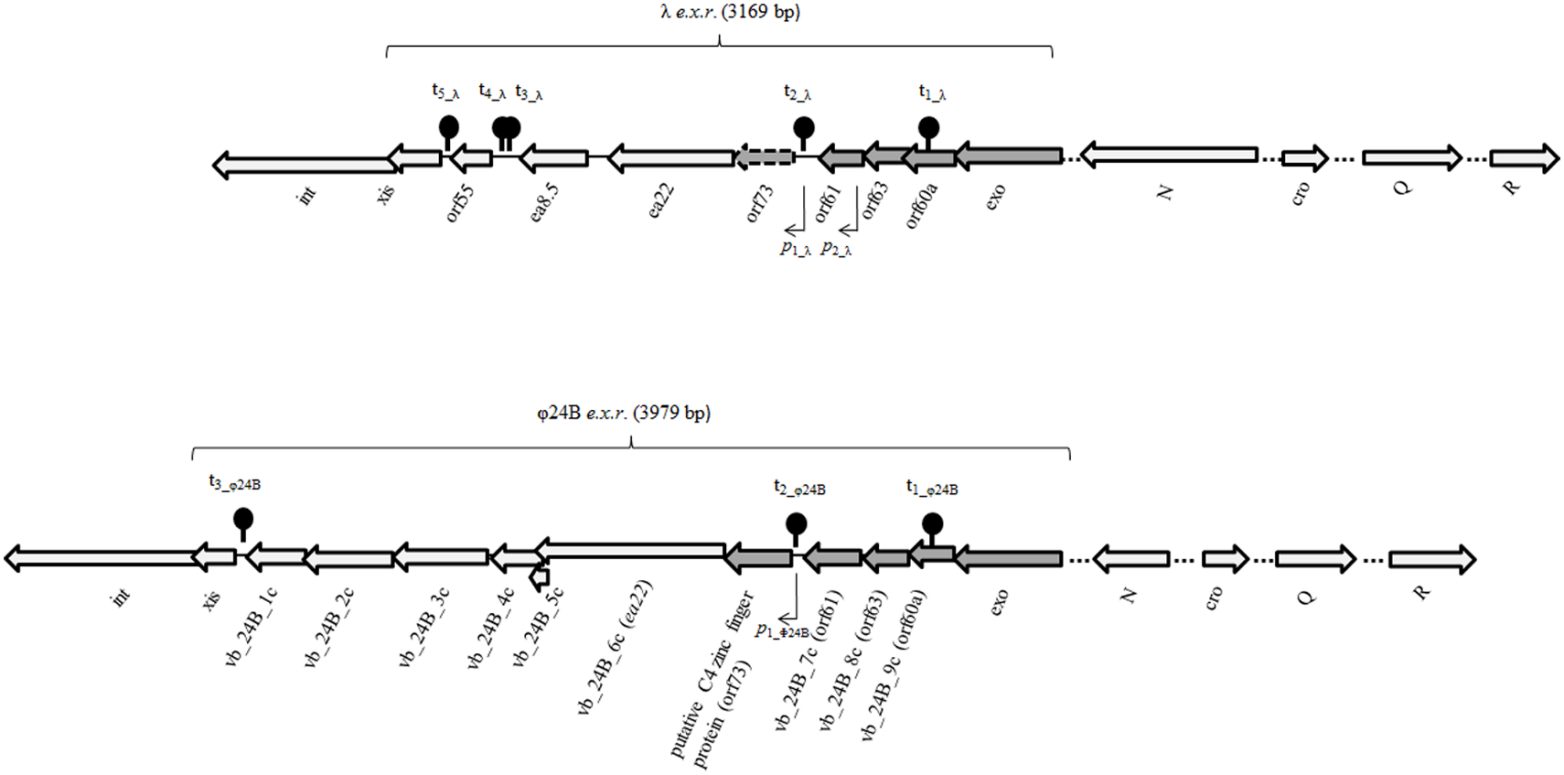
Maps of *exo-xis* regions and other genes of bacteriophages λ and Ф24_B_ analyzed in this work (accession numbers: GI:9626243 and GI:307604077 respectively). Dark arrows with continuous outside lines represent highly conserved genes and open reading frames (over 70% nucleotide as well as amino acid sequence identity). Dark arrows with punctuated outside lines represent highly conserved (above 70% sequence identity) open reading frames present in genomes of λ and 933W phages, available in the NCBI database but uncharacterized in annotations. The presence of *orf73* in the λ *exo-xis* region was indicated by [48]. Light arrows represent genes and open reading frames with low level of sequence identity (<38%). Note the high homology between λ and Ф24_B_ *exo-orf73* regions and low level of identity of other analyzed genes. Arrows indicate positions of promoters predicted with BPROM program. The localizations and −10 and −35 sequences of predicted promoters *p*_1_λ_ and *p*_1_Φ24B_ are exactly the same (see Table 3). Schematic steam-loop structures [•] indicate localizations of predicted transcription terminators, found on the basis of nucleotide seuquence analysis with ARNold software. The localizations and sequences of predicted terminators t_1_ and t_2_ are exactly the same in case of both phages λ and Ф24_B_ (see Table 4). Note that in the case of Ф24_B_ phage, some ORFs from the *exo-xis* region: *vb_24B_9c, vb_24B_8c, vb_24B_7c*, putative C4 zinc finger protein and *vb_24B_6c* are homologues of λ *orf60a, orf63, orf61, orf73* and gene *ea22* respectively. For clarity of this work, only the names of λ ORFs were used.

In the light of the above facts, we aimed to investigate the *exo-xis* region in more details. Genetic maps of *exo-xis* regions from genomes of bacteriophages λ and Ф24_B_ are shown in Fig. 1. We asked what are patterns of expression of genes from this region in *E. coli* cells either infected with bacteriophage λ or Ф24_B_, or lysogenic with these phages (after prophage induction). Unexpectedly, we found that in both phages λ and Ф24_B_, these patterns were significantly different not only between conditions of infection of the host cells by bacteriophages and prophage induction, but also between induction of prophages with various agents (mitomycin C and hydrogen peroxide). This may shed a new light on our understanding of regulation of lambdoid phage development, depending on the mode of lytic cycle initiation.

## Materials and Methods

### Bacterial strains, bacteriophages, and plasmids

Phages Φ24 (Δ*stx2*::*cat*) [25] and λ papa (from our collection) were employed in this study. Bacteriophage suspensions were routinely stored in TM buffer (10 mM Tris-HCl, 10 mM MgSO_4_, pH 7.2) at 4°C. *E. coli* MG1655 strain [26] was the host of choice for bacteriophage infection and prophage induction experiments. Plasmids pGAW3775tet (bearing phage λ *exo-xis* region), pSBe.x.r φ24B (as pGAW3775tet but bearing the *exo-xis* region from phage φ24_B_), and pJW0tet (pGAW3775tet with removed λ *exo-xis* region), used in this work, have been described [23]. Derivatives of pGAW3775, constructed previously [22] and bearing various fragments of the λ *exo-xis* region, are as follows: pJWea8.5 (pJW0tet bearing the *ea8.5* gene), pJWea22 (pJW0tet bearing the *ea22* gene), pJWorf (pJW0tet bearing *orf61*, *orf60a* and *orf63* open reading frames), pJWorfea22 (pJW0tet bearing *orf61*, *orf60a* and *orf63* open reading frames and the *ea22* gene), and pJWea22ea8.5 (pJW0tet bearing *ea22* and *ea8.5* genes). The frameshift mutations in each one of the analyzed ORFs (*orf60a, orf63, orf61, orf73*) and genes (*ea22, ea8.5*) within the λ *exo-xis* region of pGAW3775tet plasmid were introduced separately by deleting one base pair, to produce plasmids pGAWorf60a_mut, pGAWorf63_mut, pGAWorf61_mut, pGAWorf73_mut, pGAWea22_mut, and pGAWea8.5_mut, respectively. The site-directed mutagenesis was performed using GeneArt Site-Directed Mutagenesis PLUS System and AccuPrime Pfx polymerase, purchased from Life Technologies, and according to the manufacturer’s protocol. Primers used in the mutagenesis, with indication of the deleted nucleotide relative to the wild-type allele, are listed in Table 1.

**Table 1:** Primers used for site-directed mutagenesis.

Primer name	Sequence (5′→ 3′)	Deleted nucleotide
**pF_pGAW3775tet_mut_orf60a**	CAATCACTTTCGTCT*CTCCGTTACAAAGCGAG	[A]
**pR_pGAW3775tet_mut_orf60a**	CTCGCTTTGTAACGGAGAGACGAAAGTGATTG	
**pF_pGAW3775tet_mut_orf63**	CAAAGCATCTTCTGTT*AGTTAAGAACGAGTATC	[G]
**pR_pGAW3775tet_mut_orf63**	GATACTCGTTCTTAACTAACAGAAGATGCTTTG	
**pF_pGAW3775tet_mut_orf61**	CTTCATATTCTGTGTG*TTATGCTTGCCGACAT	[C]
**pR_pGAW3775tet_mut_orf61**	ATGTCGGCAAGCATAACACACAGAATATGAAG	
**pF_pGAW3775tet_mut_orf73**	GAAATAGAAGAATTAC*GCGCAACACAGCAATAA	**[A]**
**pR_pGAW3775tet_mut_orf73**	**TTATTGCTGTGTTGCGCGTAATTCTTCTATTTC**	
**pF_pGAW3775tet_mut_ea22**	TGGGGATTTGACGCAG*CCTTTTCCATGAATTG	[A]
**pR_pGAW3775tet_mut_ea22**	CAATTCATGGAAAAGGCTGCGTCAAATCCCCA	
**pF_pGAW3775tet_mut_ea8.5**	TTATCAATGTTGTGCAG*TCCGGTGTCTTGTCTC	[A]
**pR_pGAW3775tet_mut_ea8.5**	GAGACAAGACACCGGACTGCACAACATTGATAA	

The asterisk (*) indicate position of deleted nucleotide in forward primer. Analogous deletion was introduced in the reverse primer but is not shown for clarity of presentation.

### Prophage induction experiments

Bacteria lysogenic with tested phages were cultured in Luria–Bertani (LB) medium at 30°C to A_600_ of 0.1. Three induction conditions were tested: 0.2 µg/ml mitomycin C, 1 mM H_2_O_2_, and UV irradiation (50 J/m^2^). At indicated times after induction, samples of bacterial cultures were harvested, and 30 µl of chloroform were added to 0.5 ml of each sample. The mixture was vortexed and centrifuged for 5 min in a microcentrifuge. Then, serial dilutions were prepared in TM buffer (10 mM Tris–HCl, 10 mM MgSO_4_; pH 7.2). Phage titer (number of phages per ml) was determined by spotting 2.5 µl of each dilution of the phage lysate on a freshly prepared LB agar (1.5%) or LB agar (1.5%) with 2.5 µg/ml chloramphenicol (according to a procedure described by [27]), with a poured mixture of 1-ml indicator *E. coli* MG1655 strain culture and 2 ml of 0.7% nutrient agar (prewarmed to 45°C), supplemented with MgSO_4_ and CaCl_2_ (to a final concentration of 10 mM each). Plates were incubated at 37°C overnight. Analogous experiments but without induction agents were performed (control experiments) with each lysogenic strain. Presented values show phage titer (PFU/ml) normalized to results of control experiments (representing ratios of phage titers in induced and non-induced cultures). Each experiment was repeated three times.

### One-step-growth experiment

Lytic development of lambdoid phages was studied in one-step-growth experiments. Bacteria were grown in LB medium supplemented with MgSO_4_ and CaCl_2_ (to a final concentration of 10 mM each) at 30°C to A_600_ = 0.2. Samples of 10 ml were withdrawn and centrifuged (3,000×*g*, 10 min). Each pellet was suspended in 1 ml (1/10 of initial volume) of 3 mM NaN_3_ in LB. Following 5-min incubation at 30°C, the phage was added to multiplicity of infection (m.o.i.) of 0.05. Phage adsorption was carried out at 30°C for 10 min. The mixture was diluted ten-fold in warm (30°C) 3 mM NaN_3_ in LB and centrifuged (3,000×*g*, 10 min). Bacterial pellet was suspended in 1 ml of LB with 3 mM NaN_3_ and centrifuged again (3,000×*g*, 10 min). This procedure was repeated three times. The suspension was then diluted 1,000-fold with LB, prewarmed to 30°C (time 0), and aerated in a water bath shaker at this temperature. The number of infective centers was estimated from nine samples taken in the interval of 0–15 min after the dilution by plating under permissive conditions. The number of intracellular progeny phages (samples previously shaken vigorously for 1 min with equal volume of chloroform and cleared by centrifugation) was estimated by plating on appropriate indicator bacteria. Plates were incubated at 37°C overnight. Each experiment was repeated three times.

### Measurement of bacterial viability during prophage induction experiments

Bacteria lysogenic for tested phages were cultured in LB medium at 30**°**C to A_600_ = 0.1 with induction agent, either 0.2 µg/ml mitomycin C or 1mM H_2_O_2_, added at time zero. At indicated times after induction, samples equal to 2×10^8^ cells/ml were withdrawn and centrifuged at 10,000×*g* for 10 min. The supernatants were removed and pellets washed in 0.85% NaCl. Bacterial suspensions were stained with LIVE/DEAD *Bac*Light Bactrial Viability Kit (Molecular Probes), according to the manufacturer’s protocol. The mentioned kit utilizes mixtures of the green–fluorescent nucleic acid stain SYTO 9 and the red-fluorescent nucleic acid dye, propidium iodide. When used alone, the SYTO 9 dye stains bacteria with intact as well as damaged membranes (e.g. arising as a result caused by phage host-cell lysis). In contrast, propidium iodide penetrates only into bacteria with damaged membranes, causing a reduction in the green dye fluorescence when both stains are present. Measurements of fluorescence were performed in microplate reader using excitation wavelength = 485 nm and emission wavelengths = 530 nm for SYTO 9 dye, and 630 nm for propidium iodide dye [28]. Data were analyzed by dividing the fluorescence intensity of stained bacterial suspensions at emission = 530 nm by the fluorescence intensity at emission = 630 nm. Presented values show percent of live bacteria normalized to results of control experiment, non-induced cultures which at each time were assumed as 100% of live bacteria. Each experiment was repeated three times.

### Estimation of relative phage DNA amounts

Bacteria lysogenic for tested phages were cultured in LB medium at 30°C to A_600_ of 0.1. Induction of prophages was provoked in lysogenic bacteria by addition of mitomycin C to a final concentration of 0.2 µg/ml or H_2_O_2_ to a final concentration 1 mM. At indicated times after induction, 2-ml samples with 120 µl of chloroform were vortexed for 10 s and centrifuged in a microcentrifuge for 5 min. The supernatants were collected and filtered through a membrane filter with pore size of 0.22 µm (Sigma-Aldrich) to remove bacterial cells. Filtered samples were first treated with DNase I (20 µg/ml; Sigma-Aldrich) for 30 min at 37°C to remove any free bacterial DNA. The viral DNA was then liberated from virions using the method described by [29], with a minor modification. All DNase-treated samples were heated at 37°C for 60 min in the presence of 20 mM EDTA (Sigma-Aldrich) and 50 µg/ml proteinase K (Sigma-Aldrich). DNA was quantified by staining with Qubit dsDNA BR Assay Kit (Invitrogen), according to the manufacturer’s instructions. Concentration of phage DNA (in µg/ml) were calculated relative to analogous experiments but without induction agents (control experiments) with each lysogenic strain. Presented values show phage DNA concentration (µg/ml) normalized to results of control experiments. Each experiment was repeated three times.

### Bacteriophage infection

Host bacteria were grown to A_600_ of 0.3 at 30°C. Then, 120 ml volume was centrifuged and the pallet was washed with 30 ml of 0.85% NaCl. After centrifugation, the pallet was suspended in 36 ml of LB medium supplemented with MgSO_4_ and CaCl_2_ (to a final concentration of 10 mM each). The mixture was incubated for 30 min at 30°C and then chilled on ice. Bacteriophage lysate was added to m.o.i. of 5. Following 30 min incubation on ice, at indicated times, 1×10^9^ bacterial cultures were treated with NaN_3_ (Sigma-Aldrich) to a final concentration of 10 mM and harvested. The preparation of RNA and cDNA were performed as described in the subsequent subsection.

### Preparation of RNA and cDNA from bacteria

For the preparation of RNA, the induction of temperate bacteriophages from *E. coli* strain MG1655 was performed with mitomycin C (final concentration 0.2 µg/ml) or H_2_O_2_ (final concentration 1 mM) as described in previous subsections. To inhibit the growth of bacteria, all samples were treated with NaN_3_ (Sigma-Aldrich) to a final concentration of 10 mM. Total RNA was isolated from 1×10^9^ bacterial cells with the High Pure RNA Isolation Kit (Roche Applied Science). RNA preparations were repeatedly digested with TURBO DNase from TURBO DNA-*free* Kit (Life Technologies) for 60 min at 37°C, as described by the manufacturer. To evaluate the quality and quantity of total isolated RNA, we used a NanoDrop spectrophotometer, considering the ideal absorbance ratio (1.8≤A260/A280≤2.0), and visualized the band patterns of total RNA by electrophoresis. The absence of DNA from RNA samples was controlled by PCR amplification, and by real-time PCR amplification of the all tested genes. RNA preparations were stored at −80°C for use. The preparation of cDNA from the total RNA samples (1.25 µg) was performed with Transcriptor Reverse Transcriptase and random hexamer primers (Roche Applied Science), following the instructions supplied by the manufacturer. cDNA reaction mixtures were diluted 10-fold for use in real-time PCR.

### Real-time PCR Assay

For transcriptional analysis of tested genes by quantitative real-time reverse transcription-PCR (qRT-PCR), the qRT-PCR was performed with the LightCycler 480 Real-Time PCR System (Roche Applied Science), with cDNA samples from lysogenic bacteria. Transcription rates of φ24_B_ and λ genes were compared in parallel to those of the *icdA* (according to a procedure described by [30]) or 16S rRNA housekeeping genes. Primers were developed by Primer3web version 4.0.0 and produced by Sigma-Aldrich or GENOMED. The transcriptional analysis of φ24_B_ and λ genes was performed with primers presented in Table 2. Real-time PCR amplifications were performed for 55 cycles in 20-µl reaction volumes by employing LightCycler 480 SYBR Green I Master (Roche Applied Science). Reactions were performed in Roche 96-well plates containing 10 µl 2x SYBR Green I Master Mix, 6.25 ng/µl cDNA and 200 nM of each gene-specific primer (Table 2). Relative quantification assays were performed with cDNA in an *icdA* or 16S rRNA and phage genes multiplex assay. All templates were amplified using the following program: 95**°**C for 5 min; 55 cycles of 95**°**C for 10 s; 60**°**C for 15 s and 72**°**C for 15 s. No template control was included with each run. Each reaction was repeated three times and the specificity of amplified products was examined by melting curve analysis immediately after the final PCR cycle, and confirmed by gel electrophoresis.

**Table 2:** Primers used in the real-time PCR assay.

Primer name	Sequence (5′→ 3′)
pF_Φ24B_int	CAGTTGCCGGTATCCCTGT
pR_Φ24B_int	TGAGGCTTTCTTGCTTGTCA
pF_Φ24B_ea22	TCAGCAACATGGCATTCACT
pR_Φ24B_ea22	GGTTGGGAAGCTGAGAGTTG
pF_Φ24B_orf73	CGAACCTCTCTGTTTACTGATAAGC
pR_Φ24B_orf73	TTCAGGGTTGTCGGACTTGT
pF_Φ24B_orf61	TTAGCCTGACGGGCAATG
pR_Φ24B_orf61	CCGACATGGGACTTGTTCA
pF_Φ24B_orf63	GGGTCTCTCTCGTTTGCTTC
pR_Φ24B_orf63	TAGCCACATCCCTTTCACAA
pF_Φ24B_orf60a	CATACAGCCCCTCGTTTAT
pR_Φ24B_orf60a	CCGAAATCCACTGAAAGCAC
pF_Φ24B_N	AGGCGTTTCGTGAGTACCTT
pR_Φ24B_N	TTACACCGCCCTACTCTAAGC
pF_Φ24B_cro	CGAAGGCTTGTGGAGTTAGC
pR_Φ24B_cro	GTCTTAGGGAGGAAGCCGTT
pF_Φ24B_Q	GGGAGTGAGGCTTGAGATGG
pR_Φ24B_Q	TACAGAGGTTCTCCCTCCCG
pF_Φ24B_R	GGGTGGATGGTAAGCCTGT
pR_Φ24B_R	TAACCCGGTCGCATTTTTC
pF_λ_int	TTTGATTTCAATTTTGTCCCACT
pR_λ_int	ACCATGGCATCACAGTATCG
pF_λ_ea8.5	GGGCAAGTATCGTTTCCACC
pR_λ_ea8.5	GCAATGTGCGAGAAATGACTG
pF_λ_ea22	GCAGTTCCAGCACAATCGAT
pR_λ_ea22	AATGCATGACGACTGGGGAT
pF_λ_orf73	CACTTCGAACCTCTCTGTTTACTG
pR_λ_orf73	CAGGGTTGTCGGACTTGTG
pF_λ_orf61	TTAGCCTGACGGGCAATG
pR_λ_orf61	CCGACATGGGACTTGTTCA
pF_λ_orf63	ACCTGGTTTCTCTCATCTGCT
pR_λ_orf63	GTTAGCCGCATCCCTTTCAC
pF_λ_orf60a	GCATACAGCCCCTCGTTTAT
pR_λ_orf60a	CCGAAATCCACTGAAAGCAC
pF_λ_N	CTCGTGATTTCGGTTTGCGA
pR_λ_N	AAGCAGCAAATCCCCTGTTG
pF_λ_cro	ATGCGGAAGAGGTAAAGCCC
pR_λ_cro	TGGAATGTGTAAGAGCGGGG
pF_λ_Q	TTCTGCGGTAAGCACGAAC
pR_λ_Q	TGCATCAGATAGTTGATAGCCTTT
pF_λ_R	ATCGACCGTTGCAGCAATA
pR_λ_R	GCTCGAACTGACCATAACCAG
pF_E.coli_icdA	CGAAGCGGCTGACTTAATTG
pR_E.coli_icdA	GTTACGGTTTTCGCGTTGAT
pF_E.coli_16SrRNA	CCTTACGACCAGGGCTACAC
pR_E.coli_16SrRNA	TTATGAGGTCCGCTTGCTCTC

### Real-time PCR data analysis

To analyze the relative changes in gene expression revealed by quantitative Real-Time PCR experiments, the calibrator normalized relative quantification method with efficiency correction (so-called E-Method) was used. The E-Method provides an efficiency corrected calculation mode by using the determined PCR efficiency of target (E_t_) as well as the efficiency of reference (E_r_). Relative fold change ratio was calculated by using the following formula, described in the application manual of Roche LightCycler Real-Time PCR Systems [31]: Normalized relative ratio = E_t_^CT(t) calibrat^°^r – CT(t) sample^/E_r_^CT(r) calibrat^°^r – CT(r) sample^ (where “t” is target, and “r” is reference).

The sample at the time point “zero” was a calibrator. The raw run data for φ24_B_ and λ genes were transferred from the LightCycler 480 to the LinRegPCR 12.5 software using the “LC480 Conversion: conversion of raw LC480 data” software (available at http://www.hartfaalcentrum.nl/index.php?main=files&sub=0). PCR efficiency was determined for each gene by LinRegPCR program [32], [33]. This software was successfully used previously to calculate PCR efficiency [34]–[38].

### Prediction of the presence of transcription promoters and terminators in phage genomes

Prediction of the presence of promoters in the sequences of genomes of λ and φ24_B_ phages was performed using BPROM – the bacterial σ^70^ promoter recognition program available at: http://linux1.softberry.com.

Promoters were searched within ∼400 bp long sequence fragment before the start of the *orf73* coding region, encompassing whole sequence of *orf61* and the region between *orf61* and *orf73*. BPROM has accuracy of *E. coli* promoter recognition about 80%, and considers promoters with score above 0.20 [39].

Predicted terminators were found within the *exo-xis* region of λ and φ24_B_ using ARNold, the online analysis tool which predicts the existence and location of rho-independent transcription terminators using RNAmotif and ERPIN complementary programs [40]–[43]. The ARNold program is available at: http://rna.igmors.u-psud.fr/toolbox/arnold/.

## Results

### Effects of the multicopy *exo-xis* region on λ and φ24_B_ development and host survival at 30°C

Previous studies demonstrated that the presence of the *exo-xis* region on a multicopy plasmid in the host cells caused enhanced efficiency of induction of prophages λ and φ24_B_ under standard laboratory conditions (rich medium, 37°C). Since the main aim of this work was to determine detailed patterns of expression of genes from the *exo-xis* regions of the tested lambdoid phages, after preliminary experiments, we decided to culture host bacteria at 30°C, rather than at 37°C, to slow metabolic processes down which made the analyzes more precise. However, such a change in cultivation conditions relative to previously reported studies (temperature 30°C instead of 37°C) made it necessary to check whether effects of multiple copies of the *exo-xis* region on phage development are similar in both experimental systems.

**Figure 2:**
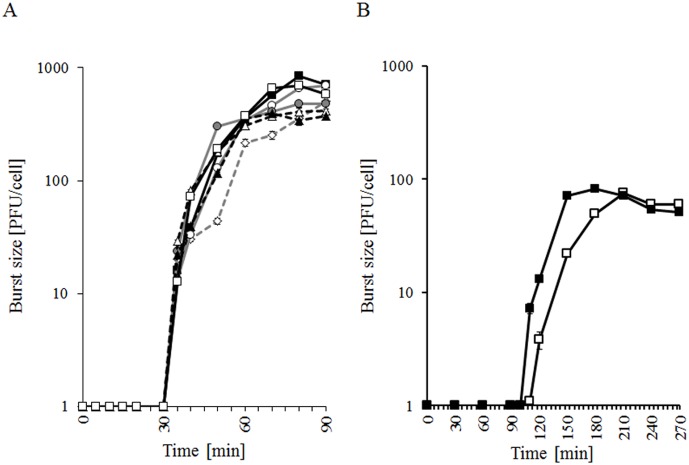
One-step-growth experiments with λ (panel A) and Ф24_B_ (panel B) bacteriophages infecting *E. coli* MG1655 host at 30°C. Host cells contained the pJW0tet vector (open squares) or a plasmid containing either the whole *exo-xis* region from λ (pGAW3773tet; panel A) or Ф24_B_ (pSBe.x.r; panel B) (closed squares), or one of following parts of this region from λ: the *ea8.5* gene (pJWea8.5; closed circles), the *ea22* gene (pJWea22; open circles), *orf61*, *orf60a* and *orf63* (pJWorf; open diamonds), *orf61*, *orf60a*, *orf63* and *ea22* (pJWorfea22; open triangles), *ea22* and *ea8.5* (pJWea22ea8.5; closed triangles). The presented results are mean values from 3 experiments with error bars indicating SD (note that in the most cases the SD were smaller than sizes of symbols).

We found that lytic developments of both tested phages, λ and φ24_B_, after infection of the *E. coli* cells growing at 30°C, were not significantly affected by the presence of corresponding *exo-xis* regions on multicopy plasmids (Fig. 2). In the case of phage λ, effects of the presence of particular fragments of this region were also tested, again with no considerable changes detected (Fig. 2). This corroborates results of analogous experiments performed at 37°C and reported previously [23].

**Figure 3:**
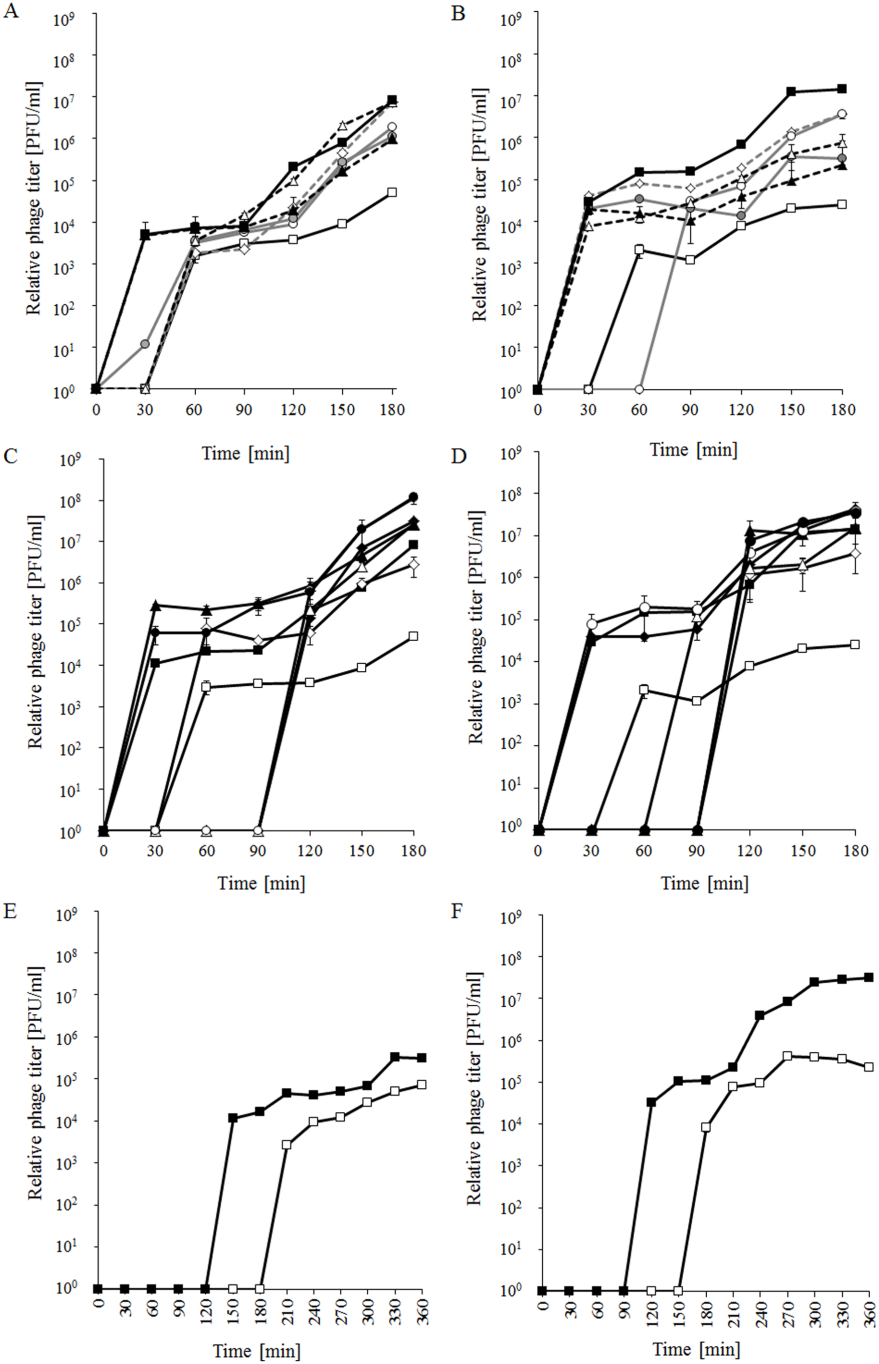
Development of λ (panels A, B, C, D) and Ф24_B_ (panels E, F) bacteriophages after prophage induction with 0.2 µg/ml mitomycin C (panels A, C, E) or 1 mM H_2_O_2_ (panels B, D, F) in *E. coli* MG1655 host at 30°C. The results with host cells containing the pJW0tet vector (open squares) or a plasmid bearing the whole *exo-xis* region from with λ (pGAW3773tet) or Ф24_B_ (pSBe.x.r) (closed squares) are presented in each panel. In other experiments presented in panels A and B, bacteria contained plasmids bearing following parts of this region from λ: the *ea8.5* gene (pJWea8.5; closed circles), the *ea22* gene (pJWea22; open circles), *orf61*, *orf60a* and *orf63* (pJWorf; open diamonds), *orf61*, *orf60a*, *orf63* and *ea22* (pJWorfea22; open triangles), *ea22* and *ea8.5* (pJWea22ea8.5; closed triangles). In other experiments presented in panels C and D, bacteria contained plasmids bearing the whole *exo-xis* region but with a frameshift mutation in one of following ORFs or genes: *orf60a* (pGAWorf60a_mut; open diamonds), *orf63* (pGAWorf63_mut; closed diamonds), *orf61* (pGAWorf61_mut; open triangles), *orf73* (pGAWorf73_mut; closed triangles), *ea22* (pGAWea22_mut; open circles) or *ea8.5* (pGAWea8.5_mut, closed circles). The presented results are mean values from 3 experiments with error bars indicating SD (note that in the most cases the SD were smaller than sizes of symbols).

Studies on λ and φ24_B_ development after prophage induction were carried out in the host cells treated with either mitomycin C or hydrogen peroxide. Again, results obtained in experiments conducted at 30°C (Fig. 3) were generally similar to those at 37°C, described in the previous article [23]. However, positive effects of multiple copies of the *exo-xis* region in both λ and φ24_B_ were even more pronounced at 30°C (compare Fig. 3 with [23]). These results also confirmed our presumption that performing the planned experiments at 30°C, rather than 37°C, may allow us to detect any putative differences more efficiently.

Accordingly to previous studies [23], we have also tested development of both tested phages in the presence of the plasmid-borne *exo-xis* region after prophage induction with UV irradiation. Similarly to experiments with mitomycin C and hydrogen peroxide (Fig. 3), production of phage progeny of both λ and φ24_B_ started earlier and was more efficient when additional copies of the *exo-xis* region were present in host cells (data not shown). Because of these similarities, in further experiments we have focused on prophage induction conditions which are more likely to occur in the intestine, a natural environment of the host bacterium [4]–[9], i.e. the presence of an antibiotic (mitomycin C) or H_2_O_2_, rather than UV irradiation.

Finding experimental conditions (30°C rather than 37°C) causing more pronounced differences in development of bacteriophages between hosts containing and lacking additional copies of the *exo-xis* region allowed us to address the problem whether expression of genes and/or ORFs from this region is responsible for the observed effects. This was reasonable as one might assume that the tested fragment of phage genome might bind regulatory factor(s), titrating it/them out, and thus causing changes is the control of viral development.

To test such a possibility, we have employed the λ model with two experimental systems. First, instead of using the whole *exo-xis* region, host cells were transformed with plasmids bearing particular genes or ORFs or their combinations. Second, we have constructed a series of derivatives of pGAW3775tet (a plasmid bearing the whole *exo-xis* region) where each plasmid contains a frameshif mutation in particular gene or ORF. In the first experimental system, the absence of particular fragments of the *exo-xis* region relative to pGAW3775tet resulted is intermediate effects, i.e. the phage development was more effective than in the host bearing the vector, but less effective than in the pGAW3775tet-bearing host (Fig. 3 A and B). However, in some cases, specific effects were detected, namely, the presence of either pJWea22 (bearing *ea22*) or pJWorfea22 (bearing *orf61*, *orf60a*, *orf63* and *ea22*) did not result in more rapid induction or phage development in the mitomycin C-treated host (Fig. 3A), and the presence pJWea22 (bearing *ea22*) caused a delay in the phage development in hydrogen peroxide-treated cells (Fig. 3B).

In the second experimental system, frameshift mutations in *orf61* and *ea22* (in the *exo-xis* fragment present in the plasmid) resulted in a delay in phage development after prophage induction with mitomycin C (Fig. 3C), while frameshit mutations in *orf61*, *orf73* and *ea8.5* caused similar effects in hydrogen peroxide-treated bacteria (Fig. 3D). In addition, we have measured the frequency of spontaneous (without addition of any specific agent) prophage induction to find similar values in cells bearing pGAW3775tet (with wild-type *exo-xis* region) and most of constructs with frameshift mutations in one of genes or ORFs (the frequencies were about 10^−5^ per cell), which were about 10 times higher than in bacteria bearing a control plasmid pJW0tet (about 10^−6^ per cell). However, a frameshift mutation in *orf60a* abolished the effect caused by the presence of a plasmid with the *exo-xis* region (the frequency was about 10^−6^ per cell). Although these results did not exclude a possibility for titrating out regulatory factors(s) by the *exo-xis* region, they suggested specific roles of expression of at least some genes and ORFs, particularly *ea22, ea8.5, orf61*, *orf73*, and *orf60a*.

**Figure 4:**
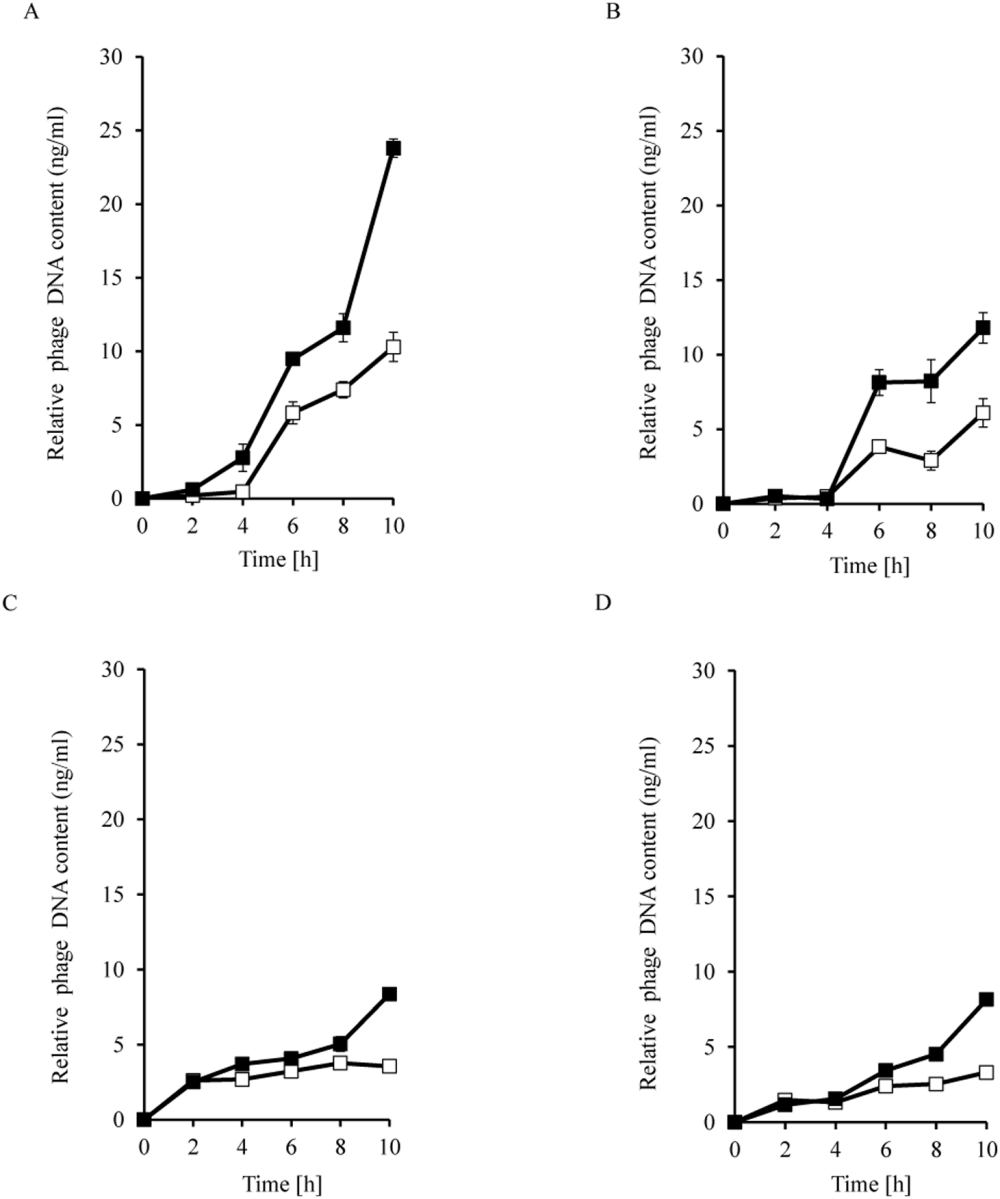
Relative level of DNA of λ (panels A and B) or Ф24_B_ (panels C and D) bacteriophages after prophage induction with 0.2 µg/ml mitomycin C (panels A and C) or 1 mM H_2_O_2_ (panels B and D) in *E. coli* MG1655 host at 30°C. Host cells contained either the pJW0tet vector (open squares) or a plasmid containing the *exo-xis* region from with λ (pGAW3773tet; panels A and B) or Ф24_B_ (pSBe.x.r; panels C and D) (closed squares). The presented results are mean values from 3 experiments with error bars indicating SD.

**Figure 5:**
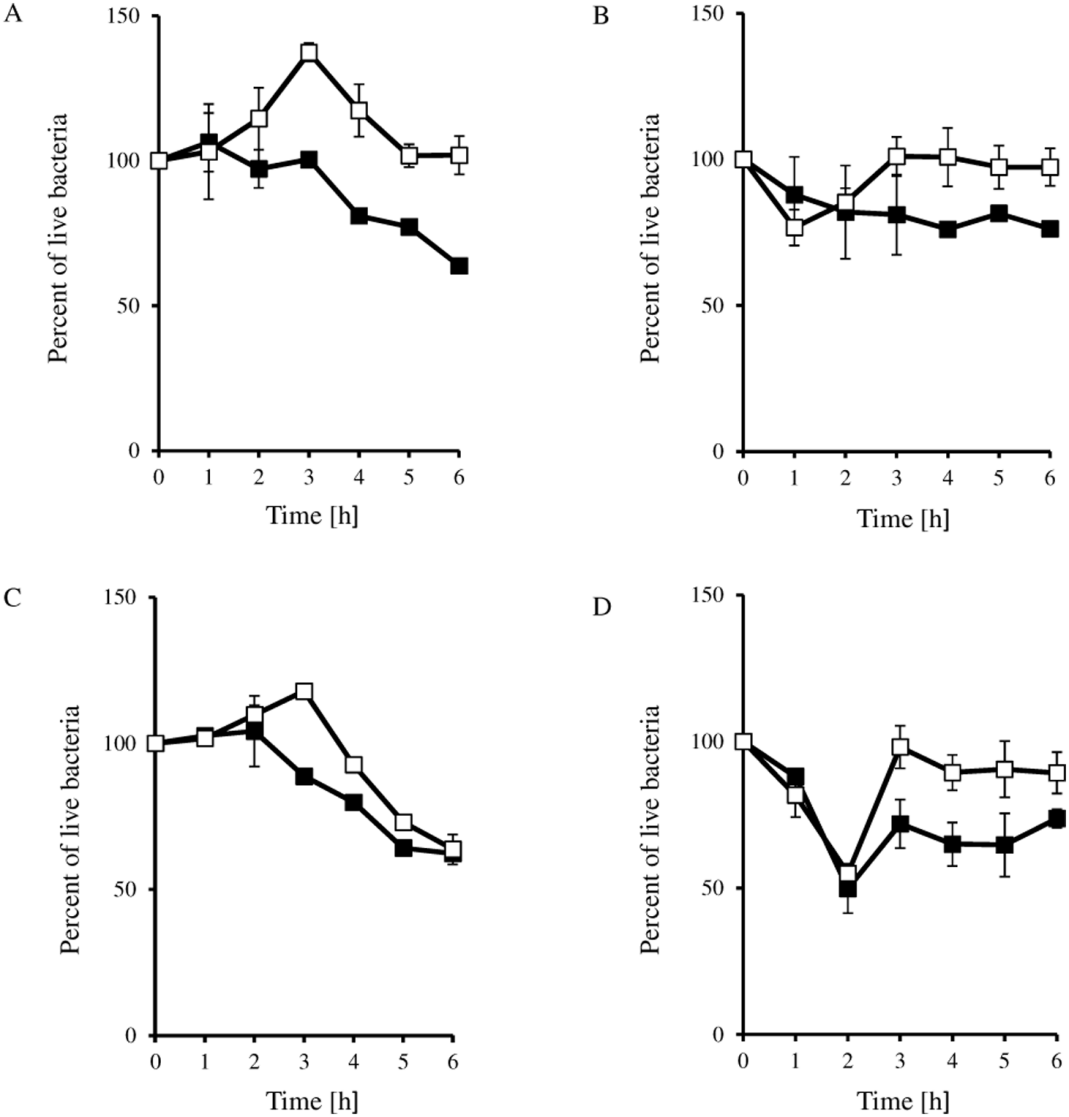
Survival of *E. coli* MG1655 cells lysogenic for λ (panels A and B) or Ф24_B_ (panels C and D) bacteriophages after prophage induction with 0.2 µg/ml mitomycin C (panels A and C) or 1 mM H_2_O_2_ (panels B and D) at 30°C. Host cells contained either the pJW0tet vector (open squares) or a plasmid containing the *exo-xis* region from with λ (pGAW3773tet; panels A and B) or Ф24_B_ (pSBe.x.r; panels C and D) (closed squares). The presented results are mean values from 3 experiments with error bars indicating SD.

Additional confirmation of the more efficient development of phages λ and φ24_B_ in cells treated at the lysogenic stage with mitomycin C or hydrogen peroxide was provided by measurement of an increase in bacteriophage DNA amount. Again, more efficient increase in the level of DNA of both tested phages was observed after induction of corresponding prophages with both tested inductors in cultures of hosts bearing plasmids with appropriate *exo-xis* region relative to those containing plasmid vector (Fig. 4). As expected, in the same experimental system, survival rate of bacteria with the vector was always higher than cells bearing a plasmid with the *exo-xis* region (Fig. 5). Survival of a high percentage of bacterial cells after the induction might seem surprising. However, one should note that contrary to UV irradiation, the efficiency of prophage induction after treatment of the host (lysogenic with λ or φ24_B_) with mitomycin C or hydrogen peroxide may be moderate. In fact, experimental data indicated that in mitomycin C- or hydrogen peroxide-treated cultures of such bacteria, prophage induction occurred in less than 50% or even in only a few percent of cells, respectively [20], [21]. Therefore, relatively large fraction of cells may survive, and due to lysogenic state, they are immune to superinfection by the same phage. This may allow these cells to grow and divide, resulting in values even over 100% in the employed experimental system (Fig. 5).

In summary, these results clearly indicated that the presence of the *exo-xis* region on a multicopy plasmid stimulates development of bacteriophages λ and φ24_B_ after treatment of lysogenic cells with either mitomycin C or hydrogen peroxide at 30°C.

### Expression of genes from the *exo-xis* regions of bacteriophages λ and φ24_B_ in infected host cells

To assess patterns of expression of genes from the *exo-xis* region in phage-infected bacteria, *E. coli* wild-type (MG1655) cells were infected with either λ or φ24_B_, and at certain times after infection total RNA was isolated and levels of tested transcripts were determined by quantitative real-time reverse transcription PCR. The *exo-xis* region is believed to be transcribed from the leftward *p*_L_ promoter, one of two major lytic promoters of lambdoid phages [3], [8]. Positions and sequences of predicted promoters and terminators located in the *exo-xis* regions of genomes of both tested phages were predicted, and they are indicated in Fig. 1 and summarized in Tables 3 and 4, respectively.

**Table 3:** Predicted promoters for the *orf73* coding region of bacteriophages λ and Ф24_B_.

Promotername	Strand	−10 box	–35 box	Promoterscore	Elements of predictedtranscriptional factorbinding sites
*p*_1_λ_	Minus	TTTTATTAT	TCATCA	4.82	rpoD17: CTCCTTT
					argR: TTTTTTAT
					argR2: TTTTTATT
*p*_1_Φ24B_	Minus	TTTTATTAT	TCATCA	4.82	rpoD17: CTCCTTT
					argR: TTTTTTAT
					argR2: TTTTTATT
*p*_2_λ_	Minus	TCATATTCT	ATGCAT	0.59	lrp: TGCATTTT
					fadR: GGACTTGT

**Table 4:** Predicted terminators in the *exo-xis* regions of bacteriophages λ and Ф24_B_.

Terminatorname	Strand	Terminatorsequence	Program thatproducedprediction	Score[Free energy ofstem-loopregion (kcal/mol)]
t_1_λ_	Minus	TTACAAAGCGA**GGCTGGG** *TATTT* **CCCGGCC**TTTCTGTTATCC	RNAmotif	−14.10
t_1_Ф24B_	Minus	TTACAAAGCGA**GGCTGGG** *TATTT* **CCCGGCC**TTTCTGTTATCC	RNAmotif	−14.10
t_2_λ_	Minus	AAAATCATCAG**GGAGCT** *ACA* **GGCTCC**TTTTTTATTATT	RNAmotif	−8.30
t_2_Ф24B_	Minus	AAAATCATCAG**GGAGCT** *ACA* **GGCTCC**TTTTTTATTATT	RNAmotif	−8.30
t_3_λ_	Minus	TTACATAACAA**TCCTCGCA** *CT* **CGCGGGGA**TTTATTTTATCTG	Erpin and RNAmotif	−11.40
t_4_λ_	Minus	TTTTATCTGAA**CTCGC** *TACG* **GCGG**GTTTTGTTTTATG	Erpin and RNAmotif	−9.20
t_5_λ_	Minus	AAGAACACCAA**GCCGCCTGATGGCGG**TTTTTTCTTGCGTG	Erpin and RNAmotif	−11.20
t_3_Ф24B_	Minus	TCAACTAACAA**CCGCC** *TTCG* **GGCGG**TTTATTATGCTG	RNAmotif	−12.70

Secondary structures are indicated, where loops are in *italic* font and stems in **bold underlined** font. The sequences of predicted terminators t_1_ and t_2_ are exactly the same in the case of both phages λ and Ф24_B_.

**Figure 6:**
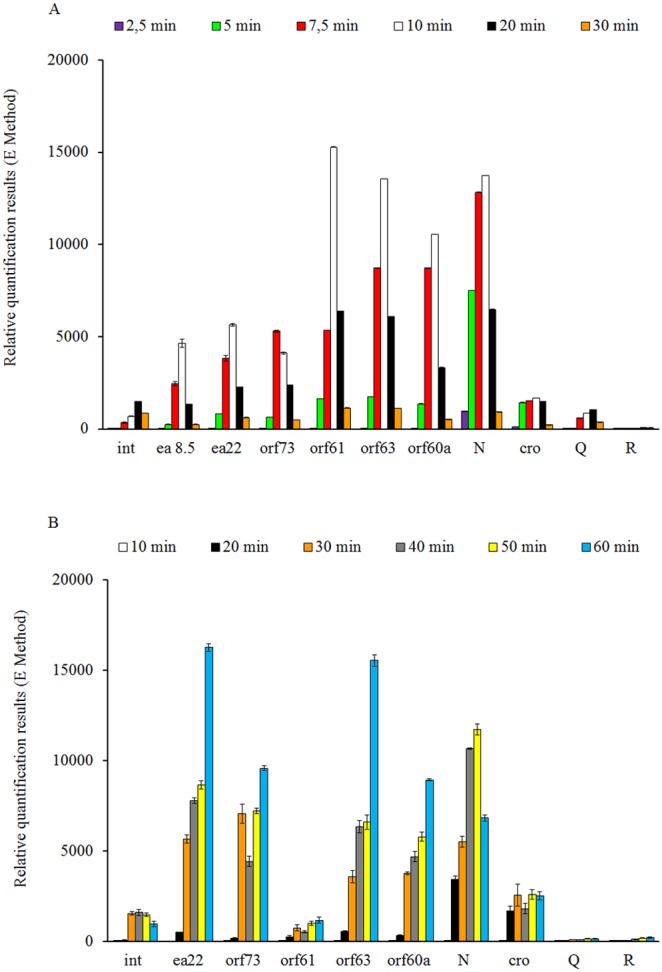
Expression patterns of genes from the *exo-xis* region, as well as *int, N, cro, Q* and *R* genes of bacteriophage λ (panel A) and Ф24_B_ (panel B) infecting *E. coli* MG1655 host at 30°C. Levels of transcripts corresponding to particular genes or *ORF*s were determined at following times after infection: 2.5 (violet), 5 (green), 7.5 (red), 10 (white), 20 (black) and 30 (orange) minutes in case of phage λ and 10 (white), 20 (black), 30 (orange), 40 (gray), 50 (yellow), and 60 (blue) minutes in case of phage 24_B_. The presented results are mean values from 3 experiments with error bars indicating SD.

Lytic development of bacteriophage λ is quicker than that of φ24_B_, as demonstrated in Fig. 3, therefore, it is not a surprise that expression of the major genes of phage lytic development, *N* and *cro*, occurred earlier after the infection with the former phage than in the latter one (Fig. 6). The presence of low level signals, followed by a maximal amount of the transcript, and finally by decreased intensities of signals in the reactions of detection of *N*- and *cro*-specific mRNAs indicates that appropriate time-frames were chosen to assess the expression efficiency. Another proof of the properly chosen times of sample withdrawn are low levels of mRNAs for *Q* and *R* genes, coding for proteins synthesized at the late stage of bacteriophage development.

Interestingly, some of the genes from the *exo-xis* regions were expressed (at the RNA level) as efficiently as, or with a similar efficiency to, the *N* gene. This was true for *orf60a, orf63,* and *orf61* in phage λ, and *orf60a, orf63, orf73,* and *ea22* in phage φ24_B_ (Fig. 6). Moreover, although levels of mRNA for *orf73, ea22* and *ea8.5*, which are downstream of the predicted terminator (located between *orf61* and *orf73*), were significantly lower than genes located upstream of the terminator in λ, considerably different expression pattern has been observed in φ24_B_. In host cells infected by the latter phage, *orf61* was poorly expressed, while levels of mRNAs for *orf73* and *ea22* were as high as those for *N, orf61a* and *orf63* (Fig. 6). Interestingly, upstream of *orf73* and *ea22*, a promoter *p*_1_Φ24B_ was predicted by BPROM program (Fig. 1 and Table 3). The *p*_1_Φ24B_ promoter sequences −10 and −35 are identical with analogous sequences of the *p*_1_λ_ promoter, localized upstream of the λ *orf73*.

### Expression of genes from the *exo-xis* regions of bacteriophages λ and φ24_B_ after prophage induction with various agents

**Figure 7:**
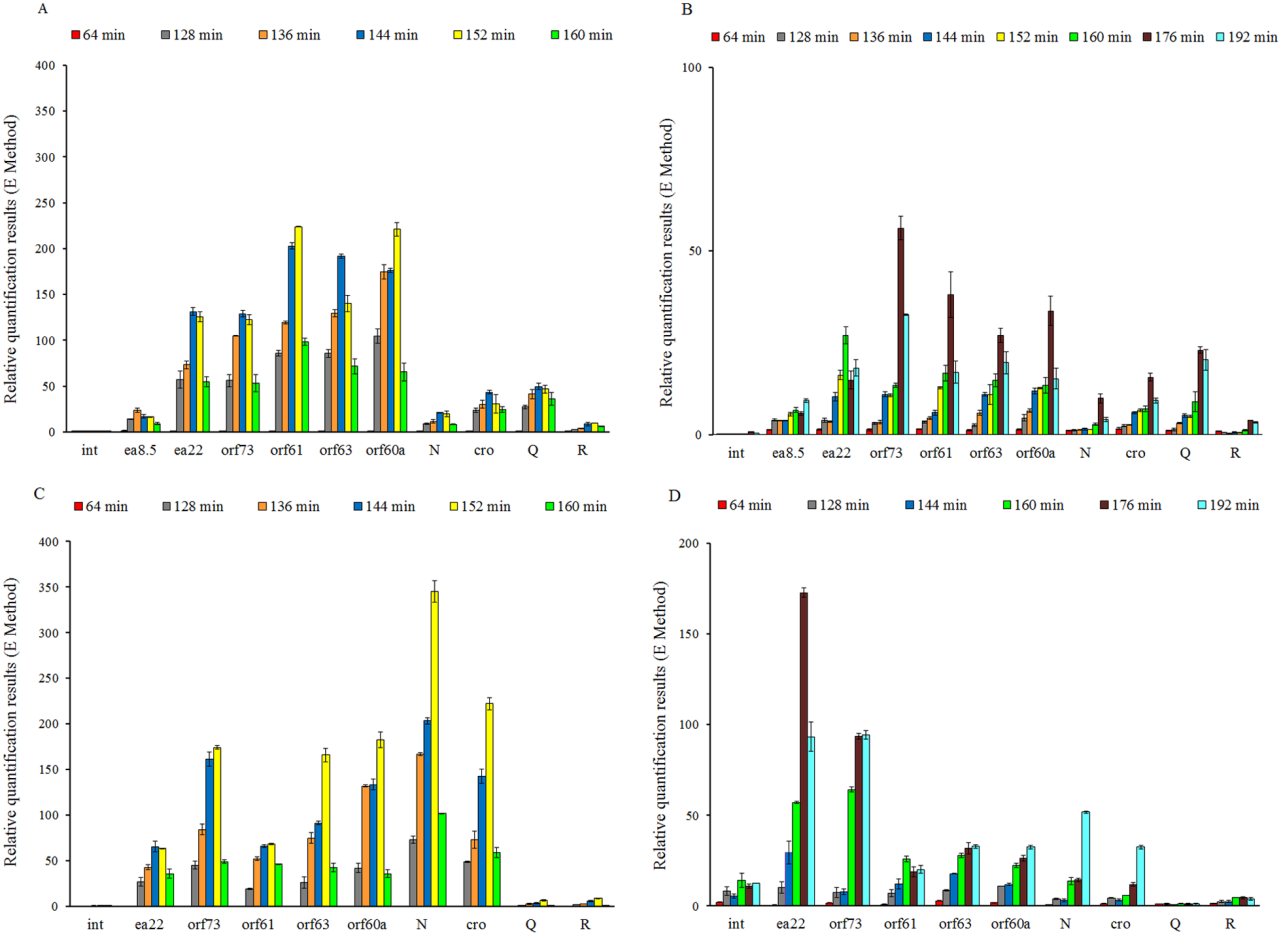
Expression patterns of genes from the *exo-xis* region, as well as *int, N, cro, Q* and *R* genes of bacteriophage λ (panels A and B) and Ф24_B_ (panels C and D) after prophage induction with 0.2 µg/ml mitomycin C (panels A and C) or 1 mM H_2_O_2_ (panels B and D) in *E. coli* MG1655 host at 30°C. Levels of transcripts corresponding to particular genes or *ORF*s were determined at following times after induction: 64 (red), 128 (gray), 136 (orange), 144 (dark blue), 152 (yellow), 160 (green), 176 (maroon) and 192 (light blue) minutes. The presented results are mean values from 3 experiments with error bars indicating SD.

Expression of phage genes after prophage induction was assessed in host cells treated with either mitomycin C or hydrogen peroxide. In both tested phages, λ and φ24_B_, characteristic time-course of mRNA amounts for *N* and *cro* genes, encompassing low level, maximum, and decreased levels, was achieved at significantly later times after induction than after infection (Fig. 7 and Fig. 6, respectively). In phage λ, levels of *N* and *cro* transcripts were significantly lower than those of genes from the *exo-xis* region, which differs from the pattern determined in phage-infected cells. Moreover, *N* and *cro* were expressed at similar times to *Q* expression, which again indicates the difference between two variants of initiation of the phage lytic development (infection vs. induction). Additionally, in the case of mitomycin C-induced λ prophage, the level of expression of *ea8.5, ea22* and *orf73* was significantly decreased in comparison with other ORFs from the *exo-xis* region (Fig. 7A) what could be explained by the presence of predicted t_2_λ_ transcription terminator, localized between *orf61* and *orf73* (Fig. 1 and Table 4).

In phage **Ф**24_B_, expression of all tested genes was delayed after the prophage induction relative to initiation of the lytic development by infection (Fig. 7 and 6, respectively). Nevertheless, expression patterns were quite similar between mitomycin C-treated lysogenic cells and bacteria infected with viruses. Surprisingly, when the prophage induction was caused by treatment of host cells with hydrogen peroxide, levels of mRNAs for *orf73* and *ea22* were significantly higher than other tested genes. This expression pattern differs considerably from that observed in mitomycin C-induced lysogens and might be explained by the presence of the *p*_1_Φ24B_ promoter upstream of *orf73* (Fig. 1 and Table 3). On the other hand, in both cases, hydrogen peroxide- and mitomycin C- induced **Ф**24_B_ prophages, the change in the expression level was observed between *orf61* and *orf73* (Fig. 7C and D). At this point, it should be noted that localizations and sequences of predicted transcription promoters *p*_1_λ_ and *p*_1_Φ24B_, as well as terminators t_2_λ_ and t_2_Φ24B_ are exactly the same (Fig. 1 and Table 4).

## Discussion

Although bacteriophage λ and related phages have been used as models for genetic studies, including regulation of gene expression, reports on global analyses of gene expression (especially time course of the expression) of lambdoid viruses are rare in the literature. Recently, expression pattern of phage λ genes after thermal induction of the prophage bearing a temeperature-sensitive mutation in the *c*I gene was investigated by ribosome profiling [44], and genes’ expression of phage φ24_B_ in the lysogenic host was studied using a proteomic approach [19]. In both cases, unexpected results were obtained. In bacteriophage λ developing after prophage induction, global gene expression analysis revealed the activity of various previously unappreciated open reading frames [44]. In both λ and φ24_B_, apart from *c*I, *rexA, rexB, lom* and *bor* – genes previously known as those transcribed from a prophage, several other genes were found to be expressed in non-induced lysogens; those included *ea8.5* in λ, and *exo* in φ24_B_, genes studied also in our work. The recently published reports, mentioned above, indicated quite an unexpected complexity in the patterns of expression of genes of lambdoid phages, suggesting the existence of multiple regulatory systems, not yet identified in these viruses.

In this work, we have investigated expression patterns of genes from the *exo-xis* regions of phages mentioned above (λ and φ24_B_) after infection of host cells or induction of corresponding prophages. We have chosen this genome region because recent studies indicated that genes included there had significant effects on efficiency of lysogenization and prophage induction in both tested phages [22], [23]. Moreover, the Ea8.5 protein, encoded in this region, was found to contain a specific motif, strongly suggesting its regulatory role [24].

Similarly to two other recent analyses [19], [44], our studies led to unexpected results. First, time courses of expression of the investigated genes, including those coding for major regulatory proteins, *N* and *cro*, were significantly different in phage-infected cells and in induced lysogens. Second, despite homologous regulatory sequences (promoters and terminators), identified and predicted in genomes of λ and φ24_B_, gene expression patterns were significantly different between these two tested phages. Third, even in the same phage, considerably different patterns of gene expression were detected, depending on the nature of agent (mitomycin C or hydrogen peroxide) used to induce the φ24_B_ prophage.

At the current stage of our knowledge, it is difficult to predict the mechanisms of the differential expression of phage genes during lytic development initiated by different ways, either infection or prophage induction. Even harder to understand is different expression of genes from the *exo-xis* region of phage φ24_B_, when prophage induction is caused by either mitomycin C or hydrogen peroxide. One would expect that both agents should induce the S.O.S. response in the host cells, which should lead to degradation of the cI repressor and subsequent prophage excision, followed by expression of phage genes as in the lytic cycle initiated by infection. Definitely, regulations of these processes are significantly more complicated than assumed.

One example of unexpected specific regulation arises from analysis of the patterns of expression of genes from the *exo-xis* region of phage φ24_B_. *In silico* analysis predicted the existence of both promoter and terminator between *orf61* and *orf73*, homologous to those in λ. In bacteriophage λ, levels of transcripts for *ORF*s located upstream of the predicted terminator are lower than those located downstream of this terminator, irrespective of the way of initiation of the lytic development. However, *orf73* and *ea22* of φ24_B_ are efficiently expressed despite the presence of this terminator, which is especially well pronounced in cells treated with hydrogen peroxide, where *orf73* and *ea22* are expressed at significantly higher levels than the rest of the *exo-xis* region. This might suggest the presence of a promoter upstream of *orf73*, and it was confirmed by the analysis with the use of BPROM program which allowed us to localize a predicted σ^70^-dependent promoter upstream of *orf73*. Despite unknown mechanisms responsible for differential expression of genes from the *exo-xis* region of bacteriophages λ and φ24_B_, the fact that transcripts of ORFs from this region occur at the significant levels during phages’ development suggests that they can play important regulatory roles in development of these viruses, as suggested previously on the basis of biological experiments [22], [23]. Such a proposal is corroborated by results presented in this report (Figs. 2–5), indicating more efficient development of both bacteriophages after prophage induction in cells bearing additional copies of the *exo-xis* region on plasmids, and by the recent finding that Ea8.5, encoded in this region, contains fused homeodomain/zinc-finger fold [24]. The experiments with plasmids bearing particular ORFs or genes, rather than the whole *exo-xis* region, as well as with plasmids bearing frameshift mutations in particular ORFs or genes, did not exclude a possibility that an excess of phage-derived sequences could cause effective binding of some regulatory factors and titrating them out. This could be responsible for observed effects on phage development. However, such a scenario seems unlikely as a sole mechanism of this phenomenon, especially in the light of specific effects of particular mutations (Fig. 3). Therefore, we suggest that it is more probable that stimulation of development of λ and φ24_B_ in cells bearing additional copies of the *exo-xis* region arises from effects of expression of certain genes, and possible cooperative actions of at least some of their products. In fact, only two genes from this region, *ea8.5* and *ea22*, were confirmed to date to encode proteins, thus others are named as *orf*s. However, specific effects of frameshift mutations in *orf61*, *orf73*, and *orf60a* (Fig. 3C and D) suggest that they may code for biologically active polypeptides.

Recent bioinformatics and microarray analyses have indicated a growing number of genes encoding small proteins in the range of 20–130 amino acids [45]–[47]. Increasing amount of experimental data demonstrates that such small proteins have variety of roles and different mechanisms of action. They can regulate functions of larger proteins, act as signaling factors or structural proteins [47]. Expression patterns of the λ genome from ribosome profiling [44] as well as our results from qRT-PCR analyses show increased expression of ORFs of unknown function during λ phage lytic development (in between these localized in the *exo-xis* region as shown in Fig. 1 from work [44] and Fig. 7 in this work). Although expression is observed at different times after induction, it could be explained by application of different inductors, temperatures of cultivation and measurement methods. Additionally, in this work we first present the increased expression of ORFs from *exo-xis* region during lytic development of phage φ24_B_. It is important to note that the level of expression of some of the analyzed ORFs from the *exo-xis* region is comparable to that of known genes or even higher. As suggested previously [44] such observation allow to suppose that these ORFs might be translated into active polypeptide products. Therefore, our further research will focus on determination of biological and biochemical roles of products of genes included in the *exo-xis* region, as well as on determination of regulatory mechanisms operating in the process of the controlling of expression of these genes.
